# The High-Molecular-Weight Glutenin Subunits of the *T. timopheevii* (A^u^A^u^GG) Group

**DOI:** 10.3390/genes15080986

**Published:** 2024-07-26

**Authors:** Benedetta Margiotta, Giuseppe Colaprico, Marcella Urbano, Daniela Panichi, Francesco Sestili, Domenico Lafiandra

**Affiliations:** 1 National Research Council, Institute of Biosciences and BioResources, 70126 Bari, Italy; margiottabenedetta@hotmail.com (B.M.); pinocolaprico@alice.it (G.C.); marcella.urbano@ibbr.cnr.it (M.U.); 2 Department of Agriculture and Forest Sciences, University of Tuscia, 01100 Viterbo, Italy; daniela.panichi76@gmail.com (D.P.); francescosestili@unitus.it (F.S.)

**Keywords:** *T. timopheevii*, gluten, high-molecular-glutenin subunits, technological quality

## Abstract

Polyploid wheats include a group of tetraploids known as Timopheevii (A^u^A^u^GG), which are represented by two subspecies: *Triticum timopheevii* ssp. *timopheevii* (cultivated) and *Triticum timopheevii* ssp. *araraticum* (wild). The combined use of electrophoretic (SDS-PAGE) and chromatographic (RP-HPLC) techniques carried out on high-molecular-weight glutenin subunits (HMW-GSs) permitted the association of different x- and y-type subunits to the A and G genomes and the assessment of allelic variation present at corresponding loci. The results also revealed that in both subspecies, accessions are present that possess expressed y-type subunits at the *Glu-A1* locus. Genes corresponding to these subunits were amplified and amplicons corresponding to x- and y-type genes associated with the A genome were detected in all accessions, including those without expressed x- and y-type subunits. The comparison with genes of polyploid wheats confirmed the structural characteristics of typical y-type genes, with the presence of seven cysteine residues and with hexapeptide and nonapeptide repeat motifs. The identification of wild and cultivated *T. timopheevii* with both x- and y-type glutenin subunits at the *Glu-A1* and *Glu-G1* loci represents a useful source for the modification of the allelic composition of HMW-GSs in cultivated wheats with the ultimate objective of improving technological properties.

## 1. Introduction

In the genus *Triticum*, wheats are divided into two groups: the emmer group with the genomic formula AABB (*Triticum turgidum* L., 2n = 4x = 28, AABB) and the *timopheevii* group with the genomic formula A^u^A^u^GG (*T. timopheevii* Zhuk., 2n = 4x = 28, A^u^A^u^GG). Both groups include a wild species: *T. dicoccoides* in the former and *T. araraticum* in the latter. *T. araraticum* (Jakubz.), well adapted to extremely dry climates and high elevations [1], is found in Armenia, Azerbaijan, Iran, Iraq, and Turkey [2]. In contrast, the cultivated subspecies *T. timopheevii* ssp. *timopheevii* was discovered by Zhukovskyi [3] in a small area of Western Georgia, where it is still grown by local farmers and used in the preparation of bread and cookies [4]. *Triticum timopheevii* has been referred to as a separate species, morphologically similar to *T. durum* but with distinctive biochemical and karyotype structures [2]. Studies on the phyletic origin of the A and G genomes have been conducted by various research groups. *T. urartu* has been identified as the A genome donor of both tetraploid wheats, *T. turgidum and T. timopheevii,* as demonstrated by studies on the variation in different repeated DNA sequences [5,6,7]. More difficulties have been encountered in the identification of the second genome of tetraploid wheats, though the independent origins of *T. timopheevii* ssp. *araraticum* and *T. turgidum* ssp. *dicoccoides* have been reported in many papers. Dvorak and Zhang [8] used a method based on the variation in repeated nucleotide sequences for the identification of diploid species most closely related to a given genome of polyploid species. Using this approach, Dvorak and Zhang [8] demonstrated that the donor of the G genome of *T. timopheevii* is a member of *Ae. speltoides*, whereas the B genome of *T. turgidum* was very likely contributed by a species in the evolutionary lineage of *Ae. speltoides*, widely separated in time and showing different times of evolutionary divergence. Additionally, the same authors suggested that the donor of the B genome to the *turgidum* group could be extinct or not yet discovered. Jang and Gill [9] supported the independent origin of *T. turgidum* and *T. timopheevii*, discovering the presence of different species-specific chromosome translocations through analyses by sequential N-banding and genomic in situ hybridization. Furthermore, the comparative analysis revealed that the genome of *Ae. speltoides* is closer to the G genome than the B genome.

The independent origins of tetraploid wheats *T. turgidum* and *T. timopheevii* were also confirmed by Rodriguez et al. [10], who, by comparing the chromosome pairing between the G and S genomes, showed a higher frequency of pairing between the G and S genomes than between the B and S genomes, thus confirming a higher degree of closeness between the G and S genomes than the B genome, indicating a more recent formation of the *timopheevii* lineage.

According to Sharma et al. [11], *T. dicoccoides* can be considered older than *T. araraticum*, with the former originating between 0.7 and 0.8 MYA and 0.4 and 0.5 MYA, whereas *T. araraticum* presumably arose between 0.1 and 0.4 MYA. Recently, Li et al. [12], using whole-genome sequences, suggested that *Ae. speltoides* diverged from the B subgenome 4.5 MYA. As the estimate that wild emmer wheat was formed 0.8 MYA [13] precludes the possibility that *Ae. speltoides* is the direct progenitor of the B subgenome, this supports the hypothesis that the donor of the bread wheat B subgenome is a diploid species closely related to, but different from, the extant *Ae. speltoides*. Similarly, it has been calculated that *Ae. speltoides* diverged from the *T. timopheevii* G subgenome ca. 2.93 MYA, following its divergence from the B subgenome progenitor. This indicates that *Ae. speltoides* could not be the direct donor of the G subgenome of *T. timopheevii* either; rather, the G subgenome of these two species was donated by a distinct diploid species of the B lineage, more closely related to the G subgenome than to the B subgenome. A subsequent hybridization of *T. timopheevii* with the diploid *T. monococcum* or *T. boeoticum* gave rise to the hexaploid *T. zhukovskyi* (2n = 6x = 42, A^u^ A^u^ A^m^A^m^GG) [14].

In bread wheat, the distinctive viscoelastic properties of dough are strongly dependent on the gluten fraction and its components, the gliadins and the glutenins, with the former providing extensibility and viscosity to the dough and the latter providing elasticity. While gliadins are monomeric proteins, glutenins are made up of polymers consisting of two groups of subunits, termed high-molecular-weight glutenin subunits (HMW-GSs) and low-molecular-weight glutenin subunits (LMW-GSs), held together by intermolecular disulfide bonds. Reduction of disulfide bonds with reducing agents releases the individual glutenin subunits, which can be separated by sodium dodecyl sulfate–polyacrylamide gel electrophoresis (SDS-PAGE).

Numerous studies have demonstrated a positive correlation between the amounts of large-sized polymers, as measured by the relative amount of unextractable polymeric proteins (%UPP), and dough properties. Gupta et al. [15] also demonstrated that the number and type of HMW-GSs play a major role in determining the amount of large-sized polymers and, indirectly, dough strength, despite HMW-GSs representing only about 10–12% of the total grain proteins [16].

In bread wheat, HMW-GSs are encoded by three orthologous pairs of genes located at the *Glu-A1*, *Glu-B1*, and *Glu-D1* loci present on the long arms of the homoeologous group 1 chromosomes of the three genomes (A, B, and D). Each locus contains two tightly linked paralogous genes, designated as x- and y-type, encoding subunits of higher and lower molecular weights, respectively [16].

Different wheat varieties show extensive polymorphism in the number (between three and five) and type of expressed HMW-GSs when separated by SDS-PAGE, caused by processes of gene silencing. In particular, both x- and y-type genes associated with the *Glu-D1* and *Glu-B1* loci are expressed, but in some varieties, silencing of the *Glu-B1y* gene has been observed. At the *Glu-A1* locus, both x- and y-type genes can be silenced, but certain varieties may have only the x-type gene expressed. The *Glu-A1y* gene is silenced in all cultivated tetraploid and hexaploid wheats as a result of a premature stop codon or WIS 2-1A retrotransposon insertion [17,18]. In addition to their major role in determining breadmaking characteristics, HMW-GSs have been used in studies associating their polymorphism, present in the wild wheat progenitor *T. dicoccoides*, with ecological and geographical parameters [19,20], whereas their value in phyletic studies has been demonstrated by Allaby et al. [21].

Unlike other species of the *Triticeae*, HMW-GSs have been studied to a very limited extent in *timopheevii* wheats. In this paper, we report the characterization of HMW-GSs present in a large number of accessions of both wild and domesticated *T. timopheevii*.

## 2. Materials and Methods

### 2.1. Plant Materials

A total of 30 accessions of *T. timopheevii* ssp. *araraticum* and *T. timopheevii* ssp. *timopheevii* were used (Table S1). The materials were obtained from the collection preserved at the National Small Grains Collection USDA-ARS. Bread wheat cultivars Cheyenne (HMW-GSs Ax2*, Bx7 + By9, Dx5 + Dy10) and Chinese Spring (Bx7 + By8, Dx2 + Dy12) and the durum wheat cultivars Duramba (Ax2*, Bx13 + By16) and Drago (Ax1, Bx6 + By8) were used as reference. A durum wheat line (IN 151/99) in which x- and y-type subunits associated at the *Glu-A1* locus from *T. dicoccoides* are present was also used (Ax21* + Ay21*, Bx7 + By8).

The seeds were divided into two sub-samples: one was used directly for RP-HPLC and SDS-PAGE analyses whereas the embryo part was saved for extraction of genomic DNA. 

### 2.2. Chromatographic Analyses by Reversed-Phase High-Performance Liquid Chromatography (RP-HPLC)

HMW-GSs were extracted following the procedure described by Marchylo et al. [22] and analyzed by RP-HPLC [23]. RP-HPLC analyses were performed on a Waters HPLC system using a Supelcosil LC-308 column with 300 Å pore size, 5 mm particle size, and 250 × 4.6 mm i.d. Separations were carried out at a solvent flow rate of 1 mL/min, using a column temperature of 65 °C, and the sample eluted was monitored at 210 nm. Solvents consisted of water and acetonitrile, each containing 0.1% (*v*/*v*) of trifluoroacetic acid. A linear gradient of 23–28% acetonitrile was used for the first 23 min, followed by a second linear gradient of 28–31% acetonitrile from 23 to 88.5 min. Fractions corresponding to peaks of RP-HPLC were collected, freeze-dried, and identified by one-dimensional electrophoresis (SDS-PAGE). 

### 2.3. Electrophoretic Analyses 

Total proteins were extracted from crushed seeds [23] with 50% propanol. The residue was suspended in 0.125 M Tris-HCl pH 6.8 buffer containing 2.75% SDS, 10% glycerol, and 0.2% DTT and incubated for 1 h at 70°C. The samples were centrifuged and 10 mL of the supernatant was analyzed by SDS-PAGE on 8% and 10% polyacrylamide gels [24,25]. 

### 2.4. N-Terminal Sequencing and MS Analyses

After SDS-PAGE electrophoresis, the proteins of interest were recovered from the gel by a cutter and put in an Eppendorf tube. The samples were analyzed at the Molecular Structure Facility Dept., Davis University CA, USA. The N-terminal of the protein of interest was determined by means of an ABI Procise 494 Edman Sequencer. Mass spectrometry analyses were performed on a TSQ Vantage Triple Quadrupole Mass Spectrometer.

### 2.5. DNA Extraction and PCR Analyses

Genomic DNA was extracted from a single seed following the modified procedure reported by Della Porta et al. [26]. The x-type genes present at the *Glu-A1* locus were amplified using the following primer pair: 5′ CCG AGA TGA CTA AGC GGT TGG TTC 3′; (b) 5′ CTG GCT GGC CAA CAA TGC GT 3′ designed on the basis of HMW glutenin subunit genes of the bread wheat variety Cheyenne [27].

DNA fragments of genes at the *Glu-A1y* locus were amplified using two primer pairs previously developed [25].

The first primer pair permitted amplification of the region lying downstream from the transposon-like insertion in the Chinese Spring allele. The second primer pair was specific to amplify the region containing the N-terminal and the repetitive domain of the *Glu-A1y* gene present in Cheyenne and at the same time to avoid the amplification of other y-type genes present on chromosomes 1B and 1D. The sequences are as follows: (a) 5′ACGTTCCCCTACAGGTACTA3′; (b) 5′TATCACTGGCTAGCCGACAA3′; (c) 5′CCATCGAAATGGCTAAGCGG3′; and (d) 5′GTCCAGAAGTTGGGAAGTGC3′.

PCR amplification was carried out in a final reaction volume of 40 μl. A template was used consisting of 50–100 ng of genomic DNA, 2.5 units of *Taq* DNA polymerase, 1 × taq PCR buffer containing the deoxyribonucleotides, and 0.1 mM of each primer. Amplification conditions were as follows: a denaturation step at 94 °C for 5 min, followed by 35 cycles at 94 °C for 1 min, 62 °C for 2 min, 72 °C for 2 min 30 s, and a final incubation step at 72 °C for 7 min. PCR amplicons were analyzed on 1% agarose gel.

## 3. Results

### 3.1. RP-HPLC Analysis of HMW-GSs Present in T. timopheevii and Araraticum Accessions 

RP-HPLC analyses of alkylated HMW-GSs resulted in effective separation and identification [23,28]; in this case, protein separation, differently from SDS PAGE, is realized on the basis of different surface hydrophobicity of glutenin subunits. A few studies of HMW-GSs present in durum and bread wheat, wild diploid, and tetraploid progenitors have clearly shown that RP-HPLC analyses of HMW-GSs provide the possibility to identify homoeoallelic subunits and differentiate between x- and y-type subunits, complementing information deduced from SDS-PAGE [23,29]. In addition, alkylation of cysteine residues with vinylpyridine influence the surface hydrophobicity of subunits with different effects on x- and y-type subunits, with the latter being eluted earlier than x-type subunits, as a consequence of modified surface hydrophobicity.

**Figure 1:**
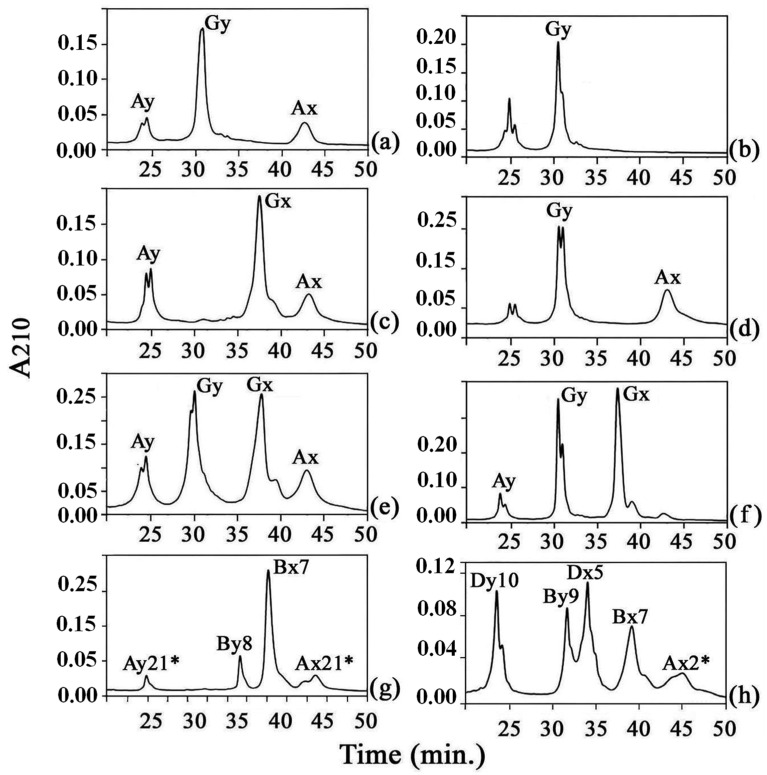
RP-HPLC of HMW-GSs present in *T. timopheevii* ssp. *araraticum* (**a**–**f**), tetraploid line (AABB) IN52/99 (**g**), and bread wheat variety Cheyenne (**h**).

In particular, the information obtained permitted us to deduce a general picture in which the elution of the subunits in bread wheat occurred in the following order: Dy, By, Dx, Bx, and Ax (Figure 1h), with minor exceptions.

Analysis of HMW-GSs present in a large collection of the tetraploid wild wheat relative *T. dicoccoides* by RP-HPLC demonstrated that in the accessions expressing four subunits, two at the *Glu-A1* and two at the *Glu-B1* locus (Figure 1g), the order of elution of the different subunits was Ay, By, Bx, and Ax [29].

RP-HPLC analysis was carried out on several accessions of *T. timopheevii* ssp. *timopheevii* and ssp. *araraticum* and a few examples of the chromatographic separations are reported in Figure 1. Based on the information available from the aforementioned different studies of the characteristics of HMW-GSs shown when separated by RP-HPLC, the first and last peaks show similar retention times of Ay and Ax subunits present in *T. dicoccoides* and the two peaks with intermediate elution order have retention times similar to By and Bx subunits (compare Figure 1e with Figure 1g) and could be assigned to the Glu-G1y and Glu-G1x, respectively. Based on the results of the chromatographic analyses, it could also be established that a variable number, from one to four subunits, is present in the different accessions analyzed. RP-HPLC also provides quantitation of different components present in a protein mixture and, in this case, the Glu-G1x and Glu-G1y subunits both show similarly high amounts, unlike that observed for the Glu-B1x and Glu-B1y subunits present in durum or bread wheat, in which the latter subunit is always present in lower quantity (Figure 1). 

Differently from what occurs in tetraploids wheats (AABB), where only the wild progenitor *T. dicoccoides* possesses the expressed Glu-A1y gene [30], in *T. timopheevii* the *Glu-A1y* gene was expressed in both the wild and the cultivated form as previously reported [31].

### 3.2. Sodium Dodecyl Sulphate Electrophoretic Separation of HMW-GSs

**Figure 2:**
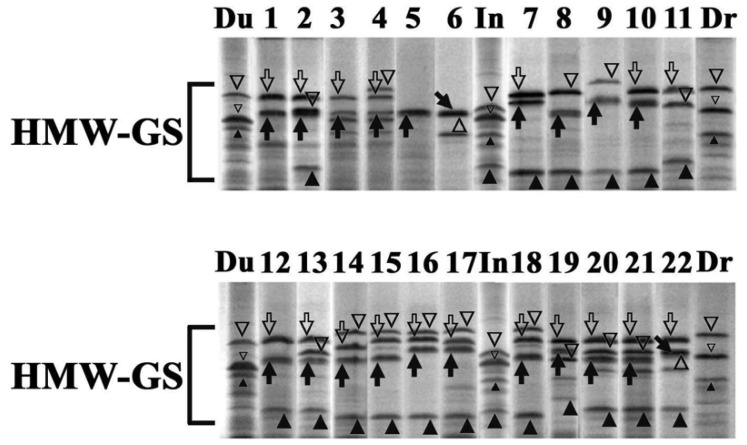
SDS-PAGE of HMW-GSs *T. timopheevii* ssp. *timopheevii* (1–2) and ssp. *araraticum* (3–22). Empty and filled arrows indicate Gx and Gy subunits, whereas empty and filled triangles indicate Ax and Ay subunits. Small triangles in the durum wheat varieties Duramba, Drago, and line IN 151/99 indicate Bx and By subunits.

The SDS-PAGE separation results of the HMW-GSs extracted from the twenty-two accessions of ssp. *timopheevii* and ssp. *araraticum* analyzed by RP-HPLC are reported in Figure 2. Assignment of different subunits to the A and G genomes is indicated. This was possible by collecting peaks after their separation by RP-HPLC and their comparison with total glutenins by SDS-PAGE separation. As evident from the electrophoretic separation, the subunits associated with the G genome show an apparent molecular weight larger than those associated with the B genome present in the durum wheat varieties Duramba, Drago, and line IN 151/99 (Figure 2).

The number of subunits varied from one (line 5) to four and Gx and Gy also showed similar staining intensity, differently from the Bx and By subunits (compare Duramba with line 1). The Ax subunits often showed apparent molecular weights lower than the homoeologous glutenin subunits associated with the G genome, in both the cultivated ssp. *timopheevii* (line 2) and wild ssp. *araraticum* (lines 11, 13, 19, 20, 21, and 22). In line 19, the Ax subunit shows an apparent molecular weight lower than the subunit Bx6 present in the durum wheat cultivar Drago. The range of variation in molecular weights resulted in smaller Ay subunits. Different apparent molecular weights showed the Gx and Gy glutenin subunits identified in the wild ssp. *araraticum*. The Gx molecular weights resulted in lower subunits than the corresponding homoeologous Ax glutenin subunits as in lines 14, 15, 16, 17, and 18, or higher as in lines 11, 13, 19, 20, 21, and 22. The mobilities of the Gx subunits range between the Ax1 present in the durum wheat cultivar Drago and the line IN 151/99 with the Ax21*. On the contrary, the Gy subunits showed a higher range of molecular weights with mobilities between the Ax2* of durum wheat cultivar Duramba and the subunit Bx7 present in IN 151/99. The accessions analyzed possessed the Gx and Gy glutenin subunits or the y-type associated with the G genome as the only expressed glutenin subunit, as in the case of line 5. 

### 3.3. PCR Analysis of x- and y-Type Genes Present at the Glu-A1 Locus in Domestic and Wild Timopheevii

**Figure 3:**
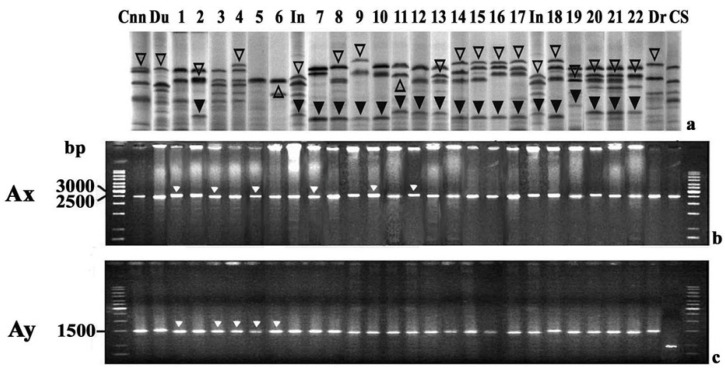
SDS-PAGE of the HMW-GSs present in the same *timopheevii* accessions reported in Figure 2 are shown and compared with the HMW-GSs present in the bread wheat variety Cheyenne (Cnn) and the durum wheat variety Duramba (Du) on the left, and the durum wheat variety Drago (Dr) and bread wheat variety Chinese Spring (Cs) on the right. (**a**,**b**) Report the DNA fragments of the *Glu-A1x* and *Glu-A1y* genes amplified by PCR. The white triangles in (**b**,**c**) indicate lines in which corresponding genes are not expressed.

The x- and y-type genes present at the *Glu-A1* locus in the 22 lines described above were analyzed by polymerase chain reaction (PCR). Amplified x-type gene fragments were detected in all the accessions. Fragments were also present in six genotypes in which the Ax-type subunits were not present, revealing that corresponding genes were silenced by the presence of SNPs or small indel mutations (lines 1, 3, 5, 7, 10, and 12 in Figure 3a,b). The control genotypes of bread and durum wheats produced an amplicon, including Chinese Spring, which is known to possess an x-type gene not expressed at the *Glu-A1* locus [25]. 

The gene encoding the Glu-A1y subunit is never expressed in bread and durum wheat; however, a rare example of an expressed *Glu-A1y* gene in bread wheat has been described by Margiotta et al. [32]. Unlike bread and durum wheats, the Ay-type gene is frequently expressed in diploid species and subspecies with the A genome as in the cultivated *T. monococcum* ssp. *monococcum* and the wild *T. monococcum* ssp. *boeoticum* and *T. urartu*. In addition, the *Glu-A1y* gene can be expressed in the wild tetraploid wheat *T. turgidum* ssp. *dicoccoides* [16] and in wild and cultivated tetraploid *T. araraticum* and *T. timopheevii*. In general, two different types of silencing were detected in the bread wheat cultivars Cheyenne and Chinese Spring. The first one has been associated with the presence of a retro transposon-like insertion (WIS-2a) of about 8 Kb, within the repetitive domain of the *Glu-A1y* gene, and was identified in Chinese Spring by Harberd et al. [18], whereas a premature stop codon resulted in the silencing of the *Glu-A1y* gene in Cheyenne [17]. Analyses on several durum and bread wheat cultivars and landraces as well as in the wild species *T. dicoccoides* have demonstrated the presence of both types of silencing *Glu-A1y* genes was due to the presence of a transposon-like insertion as reported for the bread wheat cultivar Chinese Spring [25,29,30]. To investigate if both silencing mechanisms were present in the different accessions of *T. timopheevii,* two different primer pairs were used. To identify the presence of the WIS-2a, a pair of primers capable of amplifying the region that lies downstream of the WIS-2a insertion in Chinese Spring was used, whereas the silencing associated with premature stop codon was investigated with a primer pair amplifying a fragment formed by the N-terminal and the repetitive domain of the Glu-A1y gene [25].

The results of these analyses demonstrated that in using the second pair of primers, the presence of an amplicon of about 1500 bp was obtained in all the accessions of *T. timopheevii* similar to the one present in the bread wheat variety Cheyenne. Whereas using the first pair of primers, the Glu-A1y of the bread wheat variety Chinese Spring gave an amplicon of 920 bp, but no amplicon was observed in the *T. timopheevii*, demonstrating that the WIS-2a insertion is not present in the *Glu-A1y* gene and that the silencing of the gene is, very likely, the result of the presence of a premature stop codon. 

### 3.4. Sequences of Ay, Gy, and a Partial Gx Gene

Three accessions were used to determine the complete sequences of five HMW-GSs, three Ay (PI427329, PI538442, and PI427346) and two Gy (PI538442 and PI427346). The two accessions PI538442 and PI427346 had the same amino acid sequence and only one is reported in Figure S3.

RP-HPLC separation of the HMW-GS was also carried out, as reported above, in order to assign the different subunits to the A and G genomes. The mobility of Ay and Gy subunits present in the accessions PI427346 and PI538442 were identical and RP-HPLC analyses demonstrated that elution times were also very similar. The complete DNA sequencing of the two genes revealed that the amino acidic sequences of the two subunits were identical in the two accessions PI427329 and PI538442 (Figure S1). 

The structure, composition, and organization of repeat motifs, distribution, and a number of cysteine residues of the Gy subunits are similar to the y-type subunits present in tetraploid (AABB) and hexaploid wheats (AABBDD) (Figure S2). The subunits have a peptide signal of 21 amino acids, an N-terminal domain of 104 amino acids, a degenerate sequence of 23 amino acids following the N-terminal domain, and a central repetitive domain with hexa- and nona-peptides (consensus PGQGQQ and GYYPTSLQQ). The repetitive domain of the Gy subunits shows a larger size than homoeologous By and Dy subunits as a consequence of large deletions present in these subunits. The repetitive domain ends with a tripeptide, followed by a C-terminal domain of 42 amino acids. The subunits present seven cysteine residues: five in the N-terminal domain, one in the repetitive domain, and one in the C-terminal domain as in homoeologous y-type subunits. The triplet peptide, present at the end of the repetitive region, is different from those normally found in the sequences of the HMW-GSs (GYD, GYA, and GYY), as shown in the sequence GYN. The same triplet is found in the sequences of the Gy subunits reported in the database [33]. In addition in two Gy sequences present in the database, the Asn present in the triplet is followed by an additional Asn marking the beginning of the C-terminal domain as in the By type subunits. 

The sequences of the two Ay subunits showed a signal peptide of 21 aa, an N-terminal domain of 104 amino acids, a repetitive region of 417 amino acids, and a C-terminal domain of 45 amino acids. The blast analysis of deduced proteins showed that 1Ay of PI427346 or PI538462 had a high similarity (99%) with an HMW-GS 1Ay of *T. timopheevii* ssp. *timopheevii* present in the database (GenBank accession no. CAC38046).

An insertion and a deletion of six amino acids, and a total of 14 amino acidic changes were identified in the repetitive region of the Glu-A1y subunit of accession PI427329 compared to the subunit present in PI427346 or PI538462 (Figure S1).

The two sequences are similar to those described for the Glu-A1y subunits, present in wild diploid species *Triticum boeoticum* (A^m^A^m^) and *Triticum urartu* (Luo et al., 2018). The degenerate sequence of twenty amino acid residues, preceding the repetitive domain, is identical to the one present in *T. urartu* [30], the wild diploid progenitor donor of the A genome to both AABB and AAGG tetraploid species [7]. A single amino acid difference is present in the degenerated sequences of *Triticum boeoticum* that shows threonine instead of isoleucine. The five cysteine residues are present in the N-terminal regions and different from By and Dy sequences. The cysteine residue present at the end of the repetitive domain is absent, as a result of the change in fenilalanine. The presence of an asparagine residue following the triplet GYD, at the beginning of the C-terminal domain, characterizes the two Glu-A1 subunits. An additional asparagine is present in the N-terminal region of PI427329. In addition, a change A399D is present in the accessions PI427346 and PI538442 (Figure S1). 

The presence of the asparagine residue has been associated with post-translational modification in wheat proteins by different authors [34,35,36,37] and it has been suggested that a class of cysteine endopeptidases asparaginyl endopeptidases is responsible for proteolitic processing observed. It is therefore possible to postulate that the asparagine present in both the Glu-A1y and Glu-G1y proteins undergo similar processing. 

The entire sequence of a *Gx* gene (GenBank accession no. ADI25074), present in the database, allowed us to make a comparison with our partial sequence and to detect the same structural characteristics. The three types of repeat motifs typical of x-type sequences, PGQGQQ, the hexapeptide, and a tripeptide with the consensus sequence GQQ, and the hexapeptide with the nonapeptide GYYPTSPQQ, are evident in both sequences. The three cysteine residues are also present in the N-terminal domain of the sequence, in agreement with the number of these residues in the x-type homoeoallelic subunits. The N-terminal domain consists of 86 residues preceding the degenerate sequence of 23 residues. The complete sequence of the gene confirms the higher molecular weight of the Glu-G1x subunits, as deduced from the SDS-PAGE analyses.

### 3.5. Evidence for PTM SDS-PAGE and RP-HPLC Analysis of HMW-GSs

**Figure 4:**
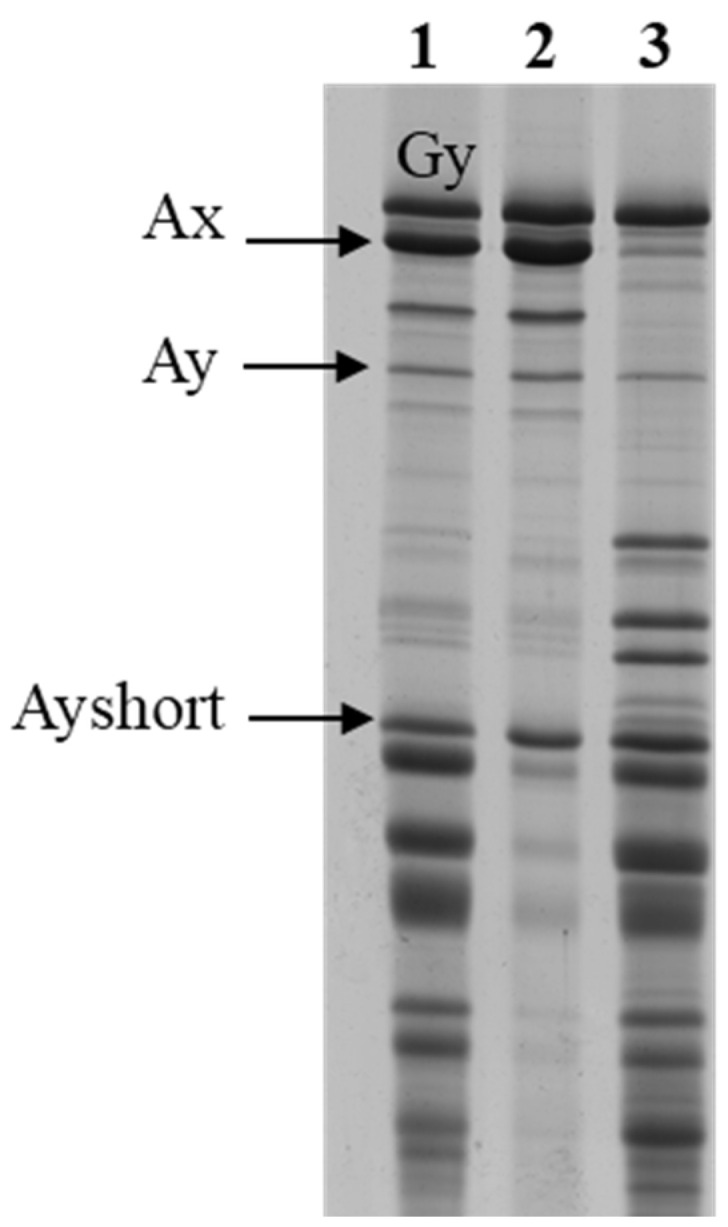
SDS-PAGE of total HMW-GSs extracted from PI427346 (lane 1), PI538442 (lane 3), and HMW-GSs precipitated from PI427346 (lane 2). The additional short component (Ays) was also present in the HMW-GSs precipitated from PI538442.

During preparation and separation by RP-HPLC of the HMW-GSs used for gene sequencing, the presence of an additional component close to the Glu-A1y subunit was detected in the two accessions of ssp. *araraticum* PI538442 and PI427346. Separation by SDS-PAGE of the same samples revealed that in addition to the HMW-GSs, an additional component (Ays) with faster mobility was present in the region of the low-molecular-weight glutenin subunits (LMW-GSs) (Figure 4). 

The additional component, present in the two *araraticum* accessions, was recovered from the gel and used for the determination of the N-terminal sequences. The result of these analyses showed that in both accessions the N-terminal sequence EGEASR, a sequence typical of the y-type HMW-GSs, was present (Figure S3). This result led us to hypothesize that the additional component could be due to a post-translational modification of the y-type HMW-GSs.

In order to gain further information on the origin of both proteins, they were analyzed by MS/MS and the results are shown in Supplementary Figure S3.

### 3.6. Mass/Mass Spectrometry Analysis

A tandem mass spectrometry analysis (MS/MS) was performed on Ay HMW-GSs present in the PI427346 and PI538442 accessions, and the two bands with reduced size associated with the two subunits. The peptides derived from the analysis of all Ay subunits were aligned (Figure S3). For Ays, all identified peptides were in the N-terminal and repetitive domains, whereas no peptides were found in the C-terminal region. In contrast, a peptide from the Glu-A1y subunit of PI538442, covering the C-terminal region, was detected (Figure S3). To predict potential cleavage sites by proteases, Glu-A1y protein sequences of the araraticum accessions were submitted to the PeptideCutter web program (http://web.expasy.org/peptide_cutter/ (accessed on 23 July 2024)). The search identified a potential site, cleaved by an Asp-N endopeptidase, which might be responsible for the production of the Ays: an aspartic acid is present at position 420 in the Glu-A1y subunits of PI427346 and PI538442 accessions, unlike PI427329, which has alanine at this position. The presence of aspartic acid in both Ay subunits suggests that Ays is the result of a cleavage of the Glu-A1y subunit. Recently, Bacala et al. [38] confirmed that an HMW-GS variant is the result of the loss of six amino acids at the C-terminus end after a conserved aspartic acid. In our case, the aspartic acid found at position 420 of the Glu-A1y subunit, present in lines PI538442 and PI427346, supports our suggestion that this residue is responsible for the generation of the Ay short protein. 

## 4. Discussion and Conclusions

Despite limited studies on *T. timopheevii*, these neglected wheats (Badaeva et al., 2021) have contributed to bread wheat improvement by providing genes that control resistance to stem and leaf rust, powdery mildew, wheat leaf blotch, black point, Hessian fly, wheat curl mite, and tan spot. *T. timopheevii* has also demonstrated resistance to abiotic stresses such as salt tolerance and excessive humidity [2,39,40]. Additionally, recent studies on both ssp. *araraticum* and ssp. *timopheevii* have highlighted the presence of nutritional and health-promoting constituents, reinforcing the value of these wheats for breeding purposes. Specifically, Hu et al. [41] analyzed twelve *T. timopheevii* ssp. *timopheevii* genotypes and found significantly higher concentrations of grain Fe and Zn compared to the bread wheat Chinese Spring and the durum wheat Langdon. Zeibig et al. [42] reported that lines of *T. araraticum* and the hexaploid *T. zhukovsky* (A^u^A^u^A^m^A^m^GG) were characterized by elevated content of Fe, protein, and antioxidant capacity, leading them to suggest that *T. araraticum* could be a good candidate for de novo domestication. Similarly, Nocente et al. [39] analyzed both ssp. *timopheevii* and ssp. *zhukovsky* for physical, nutritional, and technological characteristics, finding that both species had high protein content, antioxidant activity, and good technological and rheological performance. These results suggest that these wheats can serve as promising raw materials for the formulation of flatbreads, biscuits, and pasta. It must be stressed that these studies considered a limited number of accessions, but the results are encouraging and indicate the need for further investigations with a larger number of accessions for better characterization and identification of traits with agronomic and nutritional value.

The important role of high-molecular-weight glutenin subunits (HMW-GSs) in determining wheat processing quality is well established. Quantitative analyses have demonstrated that HMW-GSs account for about 1–1.7% of the flour dry weight. Nevertheless, they account for between 45 and 70% of the variation in breadmaking performance within European wheats [43]. Additionally, HMW-GSs can be manipulated to tailor a combination of flour protein profiles and dough-making attributes that provide desired functionality, suitable for different end products, such as wheat flour tortillas, steamed bread, noodles, and cookies [44,45,46,47].

Information on HMW-GSs present in wild and domesticated *T. timopheevii* is also very limited, and studies on genetics, diversity, and their role in the quality of end products are scarce. Difficulties in gene cloning due to their molecular size and consequent poor separation by SDS-PAGE have hindered genetic and diversity studies.

The combination of RP-HPLC and SDS-PAGE has allowed the difficulties in determining HMW-GSs present in tetraploid wheat with the genomic formula AAGG when separated by SDS-PAGE to be overcome, enabling the assignment of components to the A and G genomes. Comparative SDS-PAGE and RP-HPLC analyses of HMW-GSs have resulted in the identification of x- and y-type glutenin subunits in accessions of *T. timopheevii* (ssp. *araraticum* and ssp. *timopheevii*) and their assignment to the A and G genomes. As evident from the electrophoretic separation of the HMW-GSs, the Gx and Gy subunits exhibit slower mobility than the homoeologous Bx and By subunits, and in some cases, they overlap with Glu-A1x. Significant differences in the mobilities of Ax and Ay subunits were observed, which is a characteristic of the wild diploids *T. urartu* [48] and *T. dicoccoides* [29].

The results obtained highlighted that the HMW-GSs associated with the G genome show two main characteristics when compared to the homoeologous subunits of the B genome: higher molecular weights and similar quantities of x- and y-type subunits. In the cultivated subspecies of *T. timopheevii*, accessions have been identified in which both x- and y-type subunits associated with the *Glu-A1* locus are expressed. This contrasts with the situation in tetraploid wheats with the genomic formula AABB, where only the wild species *T. dicoccoides* possess both x- and y-type active genes at the *Glu-A1* locus [20]. Additionally, PCR results identified silent Ax- and Ay-type genes in ssp. *araraticum* and ssp. *timopheevii* and revealed the absence of the 8 kb transposon-like insertion in all accessions analyzed, contrasting with what is observed in cultivated tetraploid and hexaploid wheats [25,30], thus confirming the independent origin of AABB and AAGG species.

Sequencing of Ay and Gy subunits and a partial sequence of a silent *Gx* gene revealed that major structural characteristics, such as N- and C-terminal regions, repeat motifs, and the number and position of cysteine residues, are conserved, similar to the HMW-GSs in homoeologous genomes present in close and more distant wheat relatives. The structure of x- and y-type Gx and Gy is similar to homoeologous A, B, and D genomes, with a higher number of interspersed repeat motifs in both Gx and Gy.

The diversity of HMW-GSs is present at both *Glu-A1* and *Glu-G1* loci in ssp. *araraticum*. Therefore, ssp. *araraticum* represents a useful source that can be utilized to manipulate glutenin subunit composition in ssp. *timopheevii*, durum, and bread wheat, in order to develop material suitable for different end uses.

## Data Availability

Data will be shared upon request.

## Supplementary Materials

The following supporting information can be downloaded at https://www.mdpi.com/article/10.3390/genes15080986/s1, Figure S1: Comparison of amino acid sequences of Glu-A1y subunits present in *T*. *araraticum* PI427346 and PI427329 with those present in different species (Tu = *Triticum urartu*; Tm = *Triticum monococcum*; ARA = *Triticum araraticum*; Tt = *Triticum timopheevii*; and Td = *Triticum dicoccoides*); Figure S2: Comparison of amino acid sequences of Glu-G1y subunits present in accessions PI427346 and PI427329 with Glu-G1y and Glu-B1y; Figure S3: Alignment of amino acid peptides obtained through MS/MS analysis. Ay corresponds to the deduced protein of the accession PI427346. A11 and A47 correspond to PI427346 and PI538442. The sequences of N-terminal sequencing are highlighted in grey, cysteines in green, and aspartic acid in blue; Table S1: List of accessions.
