# Supplementary material for: The High-Molecular-Weight Glutenin Subunits of the T. timopheevii (AuAuGG) Group
Source: Genes (Basel). 2024 Jul 26;15(8):986. doi: 10.3390/genes15080986 (PMC11353860; doi:10.3390/genes15080986)
Supplement: Supplementary file 1 [file genes-15-00986-s001.zip › genes-3107442-supplementary.pdf]

```
AyTm_ACH81935      MAKRLVLFATVVIALVAFTAAEGEASRQLQCERELQESSLEACRQVVDQQLAGRLPWSTG     60
AyTu_MF568436      MAKRLVLFATVVIGLVALTVAEGEASRQLQCERELQESSLEACRLVVDQQLAGRLPWSTG     60
AyTm(e)_ACH81934   MAKRLVLFATVVIGLVALTVAEGEASRQLQCERELQESSLEACRLVVDQQLAGRLPWSTG     60
AyTu_MF568435      MAKRLVLFATVVIGLVALTVAEGEASRQLQCERELQESSLEACRLVVDQQLAGRLPWSTG     60
Ay_PI427346        MAKRLVLFATIVIGLVALTVAEGEASRQLQCERELQESSLEACRLVVDQQLAGRLPWSTG     60
AyTt_AJ306977      MAKRLVLFATVVIGLVALTVAEGEASRQLQCERELQESSLEACRLVVDQQLAGRLPWSTG     60
AyTd_KU870420      MAKRLVLFATVVIGLVSLTVAEGEASKQLQCERELQESSLEACRLVVDQQLAGRLPWSTG     60
Ay_PI427329        MAKRLVLFATVVIGLVALTVAEGEASRQLQCERELQESSLEACRLVVDQQLAGRLPWSTG     60
AyTm_JQ318694      MAKRLVLFATVVIGLVALTVAEGEASRQLQCERELQESSLEACRLVVDQQLAGRLPWSTG     60
                   **********:**.**::*.******:***************** ***************

AyTm_ACH81935      LQMRCCQQLRDVSAKCRPVAVSQVARQYEQTAVLPKGGSFYPSETTPLQQLQQVIFWGTS     120
AyTu_MF568436      LQMRCCQQLRDISAKCRPVAVSQVARQYGQTAVPPKGGSFYPRETTPLQQLQQGIFGGTS     120
AyTm(e)_ACH81934   LQMRCCQQLRDISAKCRPVAVSQVARQYGQTAVPPKGGSFYPRETTPLQQLQQGIFGGTS     120
AyTu_MF568435      LQMRCCQQLRDISAKCRPVAVSQVARQYGQTAVPPKGGSFYPRETTPLQQLQQGIFGGTS     120
Ay_PI427346        LQMRCCQQLRDISAKCRPVAVSQVARQYGQTAVPPKGGSFYPRETTPLQQLQQGIFGGTS     120
AyTt_AJ306977      LQMRCCQQLRDISAKCRPVAVSQVARQYGQTAVPPKGGSFYPRETTPLQQLQQGIFGGTS     120
AyTd_KU870420      LQTRCCQQLRDISAKCRPVALSQVARQYGQTAVPPKGGSFYHRETTSLQQLQQGIFGGTS     120
Ay_PI427329        LQMRCCQQLRDISAKCRPVAVSQVARQYGQTAVPPKGGSFYPHENTPLQQLQQGIFGGTS     120
AyTm_JQ318694      LQMRCCQQLRDISAKCRPVAVSQVARQYGQTAVPPKGGSFYSRETTPLQQLQQGIFGGTS     120
                   ** ********:********:******* **** *******  *.* ****** ** ***

AyTm_ACH81935      SQTVQGYYPSVTSPQQGSYYPGQASPQQPERGQEPGIWQEPGQGQQGYYPTSLQQSGQGQ     180
AyTu_MF568436      SQTVQGYYPSVISPQQGSYYPGQASPQQ------PGKWQELGQGQQGYYPTSLQQPGQGQ     174
AyTm(e)_ACH81934   SQTVQGYYPSVISPQQGSYYPGQASPQQ------PGKWQELGQGQQGYYPTSLQQPGQGQ     174
AyTu_MF568435      SQTVQGYYPSVISPQQGSYYPGQASPQQ------PGKWQELGQGQQGYYPTSLQQPGQGQ     174
Ay_PI427346        SQTVQGYYPSVISPQQGSYYPGQASPQQ------PGKWKELGQGQQGYYPTSLQQPGQGQ     174
AyTt_AJ306977      SQTVQGYYPSVISPQQGSYYPGQASPQQ------PGKWKELGQGQQGYYPTSLQQPGQGQ     174
AyTd_KU870420      SQTVQGYYPSVISPQQGSYYPGQASPQQ------PGKWQELGQGQQWYYPTSLQQPGQGQ     174
Ay_PI427329        SQTVQGYYPSVISPQQGSYYPGQASPQQ------PGKWQELGQGQQGYYPTSLQQPGQGQ     174
AyTm_JQ318694      SQTVQGYYPSVISPQQGSYYPGQASPQQ------PGKWQELGQGQQGYYPTSLQQPGQGQ     174
                   *********** ****************      ** *:* ***** ******** ****

AyTm_ACH81935      QGYYPSSLQQPGQGQQTGQGQQGYYPSYLQQPGQGQQIGQGQQGYYPTSPQHPGQRQQPR     240
AyTu_MF568436      QG---------------------YYRTSLQQPGQGQQIG---------------------     192
AyTm(e)_ACH81934   QG---------------------YYRTSLQQPGQGQQIG---------------------     192
AyTu_MF568435      QG---------------------YYRTSLQQPGQGQQIG---------------------     192
Ay_PI427346        QG---------------------YYRTSLQQPGQGQQIG---------------------     192
AyTt_AJ306977      QG---------------------YYRTSLQQPGQGQQIG---------------------     192
AyTd_KU870420      QGYYRTSLQQPGQ------RQQGYYRTSLQQPGQGQQIG---------------------     207
Ay_PI427329        Q---------------------GYYRTSLQQPGQGQQIG---------------------     192
AyTm_JQ318694      QGYYRTSLQQPGQ------GQQGYYRTSLQQPGQGQQIG---------------------     207
                   *                      ** : ***********

AyTm_ACH81935      QGQQIGQEQQPGQWQQGYYPTSPQQPGQGQQPGQWQQTGQGQQPKQEQQSGQGQQTGQPG     300
AyTu_MF568436      ------------QWQQGYYPTSPQHPGQGQQPGQVQKIGQGQQPEKGQQLGQEQQIG---     237
AyTm(e)_ACH81934   ------------QWQQGYYPTSPQHPGQGQQPGQVQKIGQGQQPEKGQQLGQEQQIG---     237
AyTu_MF568435      ------------QWQQGYYPTSPQHPGQGQQPGQVQKIGQGQQPEKGQQLGQEQQIG---     237
Ay_PI427346        ------------QWQQGYYPTSLQHPGQGQQPGQVQKIGQGQQSEKGQQLGQEQQIG---     237
AyTt_AJ306977      ------------QWQQGYYPTSLQHPGQGQQPGQVQKIGQGQQSEKGQQLGQEQQIG---     237
AyTd_KU870420      ------------QWQQGYYPTSPQHPGQGQQPGQVQKIGQGQQPEKGQQLGKEQQIG---     252
Ay_PI427329        ------------QWQQGYYPTSLQHPGQGQQPGQVQKIGQGQQPEKGQQLGQEQQIG---     237
AyTm_JQ318694      ------------QWQQGYYPTSPQHPGQGQQPGQVQKIGQGQQPEKGQQLGQEQQIG---     252
                               ********** *:********* *: ***** :: ** *: ** *

AyTm_ACH81935      ERQQPGQGQQTGQGQQIEQEQQSGQVQQEYYPTSPQKPGQGQQPGQSQQPGQGQQGYYPT     360
AyTu_MF568436      ------QG---------------------------QQP------GQGQQPGQGQQGYYPT     258
AyTm(e)_ACH81934   ------QG---------------------------QQP------GQGQRPGQGQQGYYPT     258
AyTu_MF568435      ------QG---------------------------QQP------GQGQQPGQGQQGYYPT     258
Ay_PI427346        ------QG---------------------------QQP------EQGQQPGQGQQGYYPT     258
AyTt_AJ306977      ------QG---------------------------QQP------EQGQQPGQGQQGYYPT     258
AyTd_KU870420      ------QG---------------------------QQPEQGQQPGQGQQPGQGQQGYYPT     279
Ay_PI427329        ------QG---------------------------QQPEQGQQPGQGQQPGQGQQGYYPT     264
AyTm_JQ318694      ------QG---------------------------QQPEQGQ------QPGQGQQGYYPT     273
                         **                           *:*          :***********

AyTm_ACH81935      SQQQQGQGQQGHYPASQQQPGQGQHGLYPTSQQQPGQGQQGHYPTSQQQAGQGQQGHYPA     420
AyTu_MF568436      SLQQPRQGQ---QPGQWQQPGQGQQGYYPTSLQQPGQGQQGHYPAS--------------     301
AyTm(e)_ACH81934   SLQQPRQGQ---QPGQWQQPGQGQQGYYPTSLQQPGQGQQGHYPAS--------------     301
AyTu_MF568435      SLQQPRQGQ---QPGQWQQPGQGQQGYYPTSLQQPGQGQQGHYPAS--------------     301
```

```
Ay_PI427346          SLQQPGQGQ---QPGQWQQAGQGQQGYYPTSLQQSGQGQQGHYPAS--------------      301
AyTt_AJ306977        SLQQPGQGQ---QPGQWQQAGQGQQGYYPTSLQQSGQGQQGHYPAS--------------      301
AyTd_KU870420        SLQQPGQGQ---QPGQWHQPGQGQQGYYPTSLQQPVQGQQGHYPAS--------------      322
Ay_PI427329          SLQQPGQRQ---QPGQWQQAGQGQQGYYPTSLQQSGQGQQGHYPAS--------------      307
AyTm_JQ318694        SPQQPRQGQ---QPGQWQQPGQGQKGYYPTSLQQPGQGQQGHYPAS--------------      316
                     * **  * *    *.. :* ****:* **** **  ********:*

AyTm_ACH81935        SLQEPGQGQQGHSPASLQQPGKGQQGHYLASLQQLGQGQQIGQPGQRQQPGQGQQIGQGQ      480
AyTu_MF568436        -QHQPGQGQQGHHPASLQQSGQGQQGHHSASLQQPGQGKQTGQREQRQQPGQGQQTGQGQ      360
AyTm(e)_ACH81934     -QHQPGQGQQGHHPASLQQSGQGQQEHHSASLQQPGQGKQTGQREQRQQPGQGQQTGQGQ      360
AyTu_MF568435        -QHQPGQGQQGHHPASLQQSGQGQQGHHSASLQQPGQGKQTGQREQRQQPGQGQQTGQGQ      360
Ay_PI427346          -QHQPGQGQQGHHPASLQQSGQGQQGHHPASLQQPGQGKQTGQREQRQQPGQGQQTGQGQ      360
AyTt_AJ306977        -QHQPGQGQQGHHPASLQQSGQGQQGHHPASLQQPGQGKQTGQREQRQQPGQGQQTGQGQ      360
AyTd_KU870420        -QHQPGQGQQGHHPASLQQSGQGQQGHHPASLQQPGQGKQTGQREQRQQPGQGQQTGQGQ      381
Ay_PI427329          -QHQPGQGQQGHHPASLQQSGQGPQGHHPASLQQPGQGKQTGQREQRQQPGQGQQTGQGQ      366
AyTm_JQ318694        -QHQPGQGQQGHHPASLQQSGQGQQGHHPASLQQPGQGKQTGQREQRQQPGQGQQTGQGQ      375
                       ::******** ****** *:* * *: ***** ***:* **  ********** ****

AyTm_ACH81935        QPEQEQQPGQGQQGYYPTYPQQPGEGQQSGQSQQPGQGQQGYYPTSLQQPGQGQQGHYPA      540
AyTu_MF568436        QPEQEQQPGQGQQGYYPTYLQQPGQGQQPEQWQQLGQGQQGHYPASLQQSGQGQQGHYPA      420
AyTm(e)_ACH81934     QPEQEQQPGQGQQGYYPTYLQQPGQGQQPEQWQQLGQGQQGHYPASLQQSGQGQQGHYPA      420
AyTu_MF568435        QPEQEQQPGQGQQGYYPTYLQQPGQGQQPEQWQQLGQGQQGHYPASLQQSGQGQQGHYPA      420
Ay_PI427346          QPEQEQQPGQGQQGYYPTYLQQPGQGQQPEQWQQPGQGQQGHYPASLQQSGQGQQGHYPD      420
AyTt_AJ306977        QPEQKQQPGQGQQGYYPTYLQQPGQGQQPEQWQQPGQGQQGHYPASLQQSGQGQQGHYPA      420
AyTd_KU870420        ------QPGQGQQGYYPTYLQQPGQGQQPEQWQQPGQGQQGHYPASLQQSGQGQQGHYPA      435
Ay_PI427329          QPEQEQQPGQGQQGYYPTYLQQPGQGQQPEQWQQPGQGQQGHYPASLQQSGQGQQGHYPA      426
AyTm_JQ318694        QPEQEQQPGQGQQGYYPTYPQQPGQGQQPEQWQQPGQGQQRHYPASLQQSGQGQQGHYPA      435
                           ************* ****:*** * ** ***** :**:**** *********

AyTm_ACH81935        SLQQPGQGHPGQRQQPGQGQQPEQEQQPGQGQEGYYPTSPQQPGQGQQLGQGQQGYYPTS      600
AyTu_MF568436        SLQQPGQGQPGQTQQPGQGQHPKQEEQPGQGQQGYYPTSPQQPGQGQQPGQGQQGHFPTS      480
AyTm(e)_ACH81934     SLQQPGQGQPGQTQQPGQGQHPEQEEQPGQGQQGYYPTSPQQPGQGQRPGQGQQGHFPTS      480
AyTu_MF568435        SLQQPGQGQPGQTQQPGQGQHPEQEEQPGQGQQGYYPTSPQQPGQGQQPGQGQQGHFPTS      480
Ay_PI427346          SLQQPGQGQPGQMQQPGQGQQPEQEQQPGQGQQGYYPTSPQQPGQGQQPGQGQQGHFPTS      480
AyTt_AJ306977        SLQQPGQGQPGQMQQPGQGQQPEQEQQPGQGQQGYYPTSPQQPGQGQQPGQGQQGHFPTS      480
AyTd_KU870420        SLQQLGQGQPGQTQQPGQGQQPEQEEQSGQGQQGYYPTSPQQPGQGQQPGQGQQGHFPTS      495
Ay_PI427329          SLQQPRQGQPGQMQQPGQGQQPEQEQQPGQGQQGYYPTSPQQPG------QGQQGHFPTS      480
AyTm_JQ318694        SLQQPGQGQPGQTQQPGQGQHPEQEEQPGQGQQGYYPTSPQQPGQGQQPGQGQQGHFPTS      495
                     ****  **:*** *******:*:**:* ****:***********      *****::***

AyTm_ACH81935        PQQPGQGQQRGQGQQRHCPTSPQQTGQAQQPGQGQQTGQVQQPGQGQQGYYPTSLQQSGQ      660
AyTu_MF568436        -------------------------GQAQQPGQGQQIGQAQQLGQGQQGYYPTSLQQPGQ      515
AyTm(e)_ACH81934     -------------------------GQAQQPGQGQQIGQAQQLGQGQQGYYPTSLQQPGR      515
AyTu_MF568435        -------------------------GQAQQPGQGQQIGQAQQLGQGQQGYYPTSLQQPGQ      515
Ay_PI427346          -------------------------GQAQQPGQGQQIGQVQQLGQGQQGYYPTSLQQPGQ      515
AyTt_AJ306977        -------------------------GQAQQPGQGQQIGQVQQLGQGQQGYYPTSLQQPGQ      515
AyTd_KU870420        -------------------------GQAQQPGQGQQIGQAQQLGQGQQGYYPTSLQQPGQ      530
Ay_PI427329          -------------------------GQAQQPGQGQQLGQVQQLGQGQQGYYPISLQQPGQ      515
AyTm_JQ318694        -------------------------GQAQQPGQGQQIGQAQQLGQGQQGYYPTSLQQPGQ      530
                                              *********** **.** ********* **** *:

AyTm_ACH81935        GQQSGQGQQSGQGHQPGQGQQSGQEQQGYNNPYHVSAEQQMASPKVAKARQPATQLPIMC      720
AyTu_MF568436        EQQSGQGQQLGQGHQPGQGQQSGQEQQGYDSPYHVSVEQQAASPKVAKAHHPVAQLPTMC      575
AyTm(e)_ACH81934     EQQSGQGQQLGQGHQPGQGQQSGQEQQGYDSPYHVSVEQQAASPKVAKAHHPVAQLPTMC      575
AyTu_MF568435        EQQSGQGQQLGQGHQPGQGQQSGQEQQGYDSPYHVSVEQQAASPKVAKAHHPVAQLPTMC      575
Ay_PI427346          EQQSGQGQQLGQGHQPGQGQQSGQEQQGYDNPYHVSVEQQAASPKVAKAHHPAAQLPIMC      575
AyTt_AJ306977        EQQSGQGQQLGQGHQPGQGQQSGQEQQGYDNPYHVSVEQQAASPKVAKAHHPAAQLPIMC      575
AyTd_KU870420        EQQSGQGQQLGQGHQPGQGQQSGQEQQGYDSPYHVSVEQQAASPKVAKAHHPVAQLPTMC      590
Ay_PI427329          EQQSGQGQQLGQGHQPGQGQQSGQEQQGYDNPYHVSMAQQAASPKVAKAHHPAAQLPTMC      575
AyTm_JQ318694        EQQSGQGQQLGQGHQPGQGQQSGQEQQGYDSPYHVSVEQQAASPKVAKAHHPVAQLPTMC      590
                      ******** ******************:.*****  ** ********::*.:*** **

AyTm_ACH81935        RMEGGEPLSASQ   732
AyTu_MF568436        QMEGGDALSASQ   587
AyTm(e)_ACH81934     QMEGGDALSASQ   587
AyTu_MF568435        QMEGGDALSASQ   587
Ay_PI427346          QMEGGDALSASQ   587
AyTt_AJ306977        QMEGGDALSASQ   587
AyTd_KU870420        QMEGGDALSASQ   602
Ay_PI427329          QMEGGDALSASQ   587
```

```
AyTm_JQ318694      QMEGGDALSASQ   602
                   :****: *****
```

Fig. S1. Comparison of amino acid sequences of Glu-A1y subunits present in *T. araraticum* PI427346 and PI427329 with those present in different species (Tu= *Triticum urartu*; Tm= *Triticum monococcum*; ARA=*Triticum araraticum*; Tt= *Triticum timopheevii*; Td= *Triticum dicoccoides*).

```
Gy_PI427346       MAKRLVLFATVVIALVAFTAAEGEASRQLQCERELQESSLEACRQVVDQQLAGRLPWSTG   60
Gy_PI427329       MAKRLVLFATVVIALVAFTAAEGEASRQLQCERELQESSLEACRQVVDQQLAGRLPWSTG   60
Gy_HM131806       MAKRLVLFATVVIALVAFTAAEGEASRQLQCERELQESSLEACRQVVDQQLAGRLPWSTG   60
Gy_EU571716       MAKRLVLFATVVIALVALTAAEGEASRQLQCERELQESPLEACRQVVDQQLAGRLPWSTG   60
Gy7*_EF151424     MAKRLVLFATVVIALVALTAAEGEASRQLQCERELQESPLEACRQVVDQQLAGRLPWSTG   60
By9_X61026        MAKRLVLFATVVITLVALTAAEGEASRQLQCERELQESSLEACRQVVDQQLAGRLPWSTG   60
By20_LN828972     MAKRLVLFATVVITLVALTAAEGEASRQLQCERELQESSLEACRQVVDQQLAGRLPWSTG   60
By15_DQ086215     MAKRLVLFATVVITLVALAAAEGEASRQLQCERELQESSLEACRQVVDQQLAGRLPWSTG   60
                  *************:***::******************* *********************

Gy_PI427346       LQMRCCQQLRDVSAKCRPVAVSQVARQYEQTAVLPKGGSFYPSETTPLQQLQQVIFWGTS   120
Gy_PI427329       LQMRCCQQLRDVSAKCRPVAVSQVARQYEQTAVLPKGGSFYPSETTPLQQLQQVIFWGTS   120
Gy_HM131806       LQMRCCQQLRDVSAKCRPVAVSQVARQYEQTAVLPKGGSFYPSETTPLQQLQQVIFWGTS   120
Gy_EU571716       LQMRCCQQLRDVSAKCRPVAVSQVARQYEQTAVLPKGGSFYPSETTPLQQLQQVIFWGTS   120
Gy7*_EF151424     LQMRCCQQLRDVSAKCRPVAVSQVARQYEQTAVLPKGGSFYPSETTPLQQLQQVIFWGTS   120
By9_X61026        LQMRCCQQLRDVSAKCRPVAVSQVVRQYEQTVVPPKGGSFYPGETTPLQQLQQVIFWGTS   120
By20_LN828972     LQMRCCQQLRDVSAKCRPVAVSQVVRQYEQTVVPPKGGSFYPGETTPLQQLQQVIFWGTS   120
By15_DQ086215     LQMRCCQQLRDVSAKCRPVAVSQVVRQYEQTVVPPKGGSFYPGETTPLQQLQQVIFWGTS   120
                  ************************.******.* ********.*****************

Gy_PI427346       SQTVQGYYPSVTSPQQGSYYPGQASPQQPEQGQEPGIWQEPGQGQQGYYPTSLQQSGQGQ   180
Gy_PI427329       SQTVQGYYPSVTSPQQGSYYPGQASPQQPEQGQEPGIWQEPGQGQQGYYPTSLQQSGQGQ   180
Gy_HM131806       SQTVQGYYPSVTSPQQGSYYPGQASPQQPEQGQEPGIWQEPGQGQQGYYPTSLQQSGQGQ   180
Gy_EU571716       SQTVQGYYPSVTSPQQGSYYPGQASPQQPEQGQEPGIWQEPGQGQQGYYPTSLQQSGQGQ   180
Gy7*_EF151424     SQTVQGYYPSVTSPQQGSYYPGQASPQQPEQGQEPGIWQGPGQGQQGYYPTSLQQSGQGQ   180
By9_X61026        SQTVQGYYPSVSSPQQGPYYPGQASPQQPGQGQQPGKWQELGQGQQGYYPTSLHQSGQGQ   180
By20_LN828972     SQTVQGYYPSVSSPQQGPYYPGQASPQQPGQRQQPGKWQELGQGQQGYYPTSLHQSGQGQ   180
By15_DQ086215     SQTVQGYYPSVSSPQQGPYYPGQASPQQPGQRQQPGKWQELGQGQQGYYPTSLHQSGQGQ   180
                  ***********:***** *********** * *:** **  ************:******

Gy_PI427346       QGYYPSSLQQPGQGQQTGQGQQGYYPSYLQQPGQGQQIGQGQQGYYPTSPQHPGQRQQPR   240
Gy_PI427329       QGYYPSSLQQPGQGQQTGQGQQGYYPSYLQQPGQGQQIGQGQQGYYPTSPQHPGQRQQPR   240
Gy_HM131806       QGYYPSSLQQPGQGQQTGQGQQGYYPSYLQQPGQGQQIGQGQQGYYPTSPQHPGQRQQPR   240
Gy_EU571716       QGYYPSSLQQPGQGQQTGQGQQGYYPSYLQQPGQGQQIGQGQQGYYPTSPQHPGQRQQPR   240
Gy7*_EF151424     QGYYPSSLQQPGQGQQTGQGQQGYYPSYLQQPGQGQQIGQGQQGYYPTSPQHPGQRQQPR   240
By9_X61026        QGYYPSSLQQPGQGQQIGQGQQGYYPTSLQQPGQGQQIGQGQQGYYPTSPQHPGQRQQPG   240
By20_LN828972     QGYYPSSLQQPGQGQQTGQGQQGYYPTSLQQPGQGQQIGQGQQGYYPTSPQHPGQRQQPG   240
By15_DQ086215     QGYYPSSLQQPGQGQQTGQGQQGYYPTSLQQPGQGQQIGQGQQGYYPTSPQHPGQRQQPG   240
                  **************** *********: *******************************

Gy_PI427346       QGQQIGQEQ------------QPGQWQQGYYPTSPQQPGQGQQPGQWQQTGQGQQPKEQ   288
Gy_PI427329       QGQQIGQEQ------------QPGQWQQGYYPTSPQQPGQGQQPGQWQQTGQGQQPKEQ   288
Gy_HM131806       QGQQIGQEQ------------QPGQWQQGYYPTSPQQPGQGQQPGQWQQTGQGQQPKEQ   288
Gy_EU571716       QEQQIGQEQ------------QPGQWQQGYYPTSPQQPGQGQQPGQWQQTGQGQQPKEQ   288
Gy7*_EF151424     QEQQIGQEQ------------QPGQWQQGYYPTSPQQPGQGQQPGQWQQTGQGQQPKEQ   288
By9_X61026        QGQQIGQGQQLGQGRQIGQGQQSGQGQQGYYPTSPQQLGQGQQPGQWQQSGQGQQGYYP-   299
By20_LN828972     QGQQIGQGQQPGQGRQIGQGQQSGQEQQGYYATSPQQLGQGQQPGQWQQSGQGQQRYYP-   299
By15_DQ086215     QGQQIGQGQQPGQGRQIGQGQQSGQEQQGYYATSPQQLGQGQQPGQWQQSGQGQQRYYP-   299
                  * ***** *            * ** ***** ***** ***********:*****

Gy_PI427346       QSGQGQQTGQPGERQQPGQGQQTGQGQQIEQEQQPGQVQQEYYPTSPQKPG---QGQQPG   345
Gy_PI427329       QSGQGQQTGQPGERQQPGQGQQTGQGQQIEQEQQPGQVQQEYYPTSPQKPG---QGQQPG   345
Gy_HM131806       QSGQGQQTGQPGERQQPGQGQQTGQGQQIEQEQQPGQVQQEYYPTSPQKPG---QGQQPG   345
Gy_EU571716       QSGQGQQTGQPGERQQPGQGQQTGQGQQIEQEQQSGQVQQEYYPTSPQKPG---QGQQPG   345
Gy7*_EF151424     QSGQGQQTGQPGERQQPGQGQQTGQGQQIEQEQQSGQVQQEYYPTSPQKPG---QGQQPG   345
By9_X61026        -----------TSQQQP------------------GQGQQGQYPASQQQPGQGQ------   324
By20_LN828972     -----------TSQQQP------------------GQGQQGQYPASQQQPAQGQQGQYPA   330
By15_DQ086215     -----------TSQQQP------------------GQGQQGQYPASQQQPAQGQQGQYPA   330
                              .:***                  ** **  **:* *:*.

Gy_PI427346       QSQQPGQGQQGYYPTSQQQQGQGQQGHYPASQQQPGQGQHGLYPTSQQQPGQGQQGHYPT   405
Gy_PI427329       QSQQPGQGQQGYYPTSQQQQGQGQQGHYPASQQQPGQGQHGLYPTSQQQPGQGQQGHYPT   405
Gy_HM131806       QSQQPGQGQQGYYPTSQQQQGQGQQGHYPASQQQPGQGQHGLYPTSQQQPGQGQQGHYPT   405
Gy_EU571716       QSQQPGQGQQGYYPTSQQQQGQGQQGHYPASQQQPGQGQHGLYPTSQQQPGQGQQGHYPT   405
Gy7*_EF151424     QSQQPGQGQQGYYPTSQQQQGQGQQGHYPASQQQPGQGQHGLYPTSQQQPGQGQQGHYPT   405
By9_X61026        ------------------------QGQYPASQQQPGQGQQGQYPASQQQPGQGQQGHYLA   360
By20_LN828972     SQQQPAQGQQGQYPASQQQPGQGQQGQYPASQQQPAQGQQGQYPASQQQPGQGQQGHYPA   390
By15_DQ086215     SQQQPAQGQQGQYPASQQQPGQGQQGQYPASQQQPAQGQQGQYPASQQQPGQGQQGHYPA   390
                                          **:********.***:* **:************* :
```

```
Gy_PI427346         SQQQAGQGQQGHYPASLQEPGQGQQGHSPASLQQPGKGQQGHYLASLQQLGQGQQIGQPG      465
Gy_PI427329         SQQQAGQGQQGHYPASLQEPGQGQQGHSPASLQQPGKGQQGHYLASLQQLGQGQQIGQPG      465
Gy_HM131806         SQQQAGQGQQGHYPASLQEPGQGQQGHSPASLQQPGKGQQGHYLASLQQLGQGQQIGQPG      465
Gy_EU571716         SQQQAGQGQQGHYPASLQEPGQGQQGHSPASLQQPGKGQQGHYLASLQQLGQGQQIGQPG      465
Gy7*_EF151424       SQQQAGQGQQGHYPASLQEPGQGQQGHSPASLQQPGKGQQGHYLASLQQLGQGQQIGQPG      465
By9_X61026          SQQQPGQGQQRHYPASLQQPGQGQQGHYTASLQQPGQGQQGHYPASLQQVGQGQQIGQLG      420
By20_LN828972       SEQQPGQGQQRHYPASLQQPGQGQQRHYAASLQQPGQGQQGHYPASLQQVGQGQQIGQPG      450
By15_DQ086215       SEQQPGQGQQRHYPASLQQPGQGQQRHYAASLQQPGQGQQGHYPASLQQVGQGQQIGQPG      450
                    *:** ***** *******:****** *  *******:****** *****:******** *

Gy_PI427346         QRQQPGQGQQIGQ------------------GQQPEQEQQPGQGQQGYYPTYPQQPGEGQ      507
Gy_PI427329         QRQQPGQGQQIGQ------------------GQQPEQEQQPGQGQQGYYPTYPQQPGEGQ      507
Gy_HM131806         QRQQPGQGQQIGQ------------------GQQPEQEQQPGQGQQGYYPTYPQQPGEGQ      507
Gy_EU571716         QRQQPGQGQQIGQ------------------EQQPEQEQQPGQGQQGYYPTYPQQPGEGQ      507
Gy7*_EF151424       QRQQPGQGQQIGQ------------------GQQPEQEQQPGQGQQGYYPTYPQQPGEGQ      507
By9_X61026          QRQQPGQGQQTRQGQQLEQGQQPGQGQQTRQGQQLEQGQQPGQGQQGYYPTSPQQSGQGQ      480
By20_LN828972       QRQQPGQGQQTEQ------------------GQQLEQGQQPGQGQQGYYPTSPQQSGQGQ      492
By15_DQ086215       QRQQPGQGQQTEQ------------------GQQLEQGQQPGQGQQGYYPTSPQQSGQGQ      492
                    **********  *                   ** ** ************* *** *:**

Gy_PI427346         QSGQSQQPGQGQQGYYPTSLQQPGQGQQGHYPASLQQPGQGHPGQRQQPGQGQQPEQEQQ      567
Gy_PI427329         QSGQSQQPGQGQQGYYPTSLQQPGQGQQGHYPASLQQPGQGHPGQRQQPGQGQQPEQEQQ      567
Gy_HM131806         QSGQSQQPGQGQQGYYPTSLQQPGQGQQGHYPASLQQPGQGHPGQRQQPGQGQQPEQEQQ      567
Gy_EU571716         QSGQSQQPGQGQQGYYPTSLQQPGQGQQGHYPASLQQPGQGHPGQRQQPGQGQQPEQEQQ      567
Gy7*_EF151424       QSGQSQQPGQGQQGYYPTSLQQPGQGQQGHYPASLQQPGQGHPGQRQQPGQGQQPEQEQQ      567
By9_X61026          QPGQSQQPGQGQQGYYSSSLQQPGQGLQGHYPASLQQPGQGHPGQRQQPGQGQQPEQGQQ      540
By20_LN828972       QPGQSQQPGQGQQGYYSTSLQQPGQGQQGHYPTSLQQPGQGHPGQRQQPGQGQQPEQGQQ      552
By15_DQ086215       QPGQSQQPGQGQQGYYSTSLQQPGQGQQGHYPTSLQQPGQGHPGQRQQPGQGQQPEQGQQ      552
                    * ************** :******** *****:************************ **

Gy_PI427346         PGQGQEGYYPTSPQQPGQGQQLGQGQQGYYPTSPQQPGQGQQ------QGQGQQRHCPTS      621
Gy_PI427329         PGQGQEGYYPTSPQQPGQGQQLGQGQQGYYPTSPQQPGQGQQ------RGQGQQRHCPTS      621
Gy_HM131806         PGQGQEGYYPTSPQQPGQGQQLGQGQQGYYPTSPQQPGQGQQ------QGQGQQRHCPTS      621
Gy_EU571716         PGQGQEGYYPTSPQQPGQGQQLGQGQQGYYPTSPQQPGQGQQ------RGQGQQRHCPTS      621
Gy7*_EF151424       PGQGQEGYYPTSPQQPGQGQQLGQGQQGYYPTSPQQPGQGQQ------RGQGQQRHCPTS      621
By9_X61026          PGQGQQGYYPTSPQQPGQGKQLGQGQQGYYPTSPQQPGQGQQPG------QGQQGHCPTS      594
By20_LN828972       PGQGQQGYYPTSPQQPGQGKQLRQGQRGYYPTSLQQPGQGQQPG------QGQQGHCPTS      606
By15_DQ086215       PGQGQQGYYPTSPQQPGQGKQLRQGQQGYYPTSLQQPGQGQQPGQGQQSGQGQQGHCPTS      612
                    *****:*************:** ***:****** ********        **** *****

Gy_PI427346         PQQTGQAQQPGQGQQTGQVQQPGQGQQGYYPTSLQQSGQGQQSGQGQQSGQGHQPGQGQQ      681
Gy_PI427329         PQQTGQAQQPGQGQQTGQVQQPGQGQQGYYPTSLQQSGQGQQSGQGQQSGQGHQPGQGQQ      681
Gy_HM131806         PQQTGQAQQPGQGQQTGQVQQPGQGQQGYYPTPLQQSGQGQQSGQGQQSGQGHQPGQGQQ      681
Gy_EU571716         PQQTGQAQQPGQGQQTGQVQQPGQGQQGYYPTSLQQSGQGQQSGQGQQSGQGHQPGQGQQ      681
Gy7*_EF151424       PQQTGQAQQPGQGQQTGQVQQPGQGQQGYYPTSLQQSGQGQQSGQGQQSGQGHQPGQGQQ      681
By9_X61026          PQQTGQAQQPGQGQQIGQVQQPGQGQQGYYPISLQQSGQGQQSGQGQQSGQGHQLGQGQQ      654
By20_LN828972       PQQTGQAQQPGQGQQIGQVQQPGQGQQGYYPISLQQSGQGQQSGQGQQSGQGHQLGQGQQ      666
By15_DQ086215       PQQTGQAQQPGQGQQIGQVQQPGQGQQGYYPISLQQSGQGQQSGQGQQSGQGHQLGQGQQ      672
                    *************** ***************  ********************* *****

Gy_PI427346         SGQEQQGYNSPYHVSAEQQMASPKVAKAQQPATQLPIMCRMEGGEPLSASQ      732
Gy_PI427329         SGQEQQGYNSPYHVSAEQQMASPKVAKAQQPATQLPIMCRMEGGEPLSASQ      732
Gy_HM131806         SGQEQQGYNSPYHVSAEQQMASPKVAKAQQPATQLPIMCRMEGGEPLSASQ      732
Gy_EU571716         SGQEQQGYNNPYHVSAEQQMASPKVAKAQQSATQLPIMCRMEGGEPLSASQ      732
Gy7*_EF151424       SGQEQQGYNNPYHVSAEQQMASPKVAKAQQSATQLPIMCRMEGGEPLSASQ      732
By9_X61026          SGQEQQGYDNPYHVNTEQQTASPKVAKVQQPATQLPIMCRMEGGDALSASQ      705
By20_LN828972       SGQEQQGYDNPYHVNTEQQTAGPKVAKVQQPATQLPIMCRMEGGDALSASQ      717
By15_DQ086215       SGQEQQGYDNPYHVNTEQQTASPKVAKVQQPATQLPIMCRMEGGDALSASQ      723
                    ********:.****.:*** *.*****.** *************: *****
```

Fig. S2. Comparison of amino acid sequences of Glu-G1y subunits present in accessions PI427346 and PI427329 with Glu-G1y and Glu-B1y.

```
Ay       MAKRLVLFATIVIGLVALTVAEGEASRQLQCERELQESSLEACRLVVDQQLAGRLPWSTGLQMRCCQQLRDISAKCRPVAVSQVARQYG
Ay11                                      ELQESSLEACRLVVDQQL   LPWSTGLQMRCCQQLR     CRPVAVSQVARQYG
Ay47                                      ELQESSLEACRLVVDQQLAGRLPWSTGLQMR           CRPVAVSQVARQYG
Ayshort47                     EGEASR      ELQESSLEACRLVVDQQLAGRLPWSTGLQMR          KCRPVAVSQVAR
Ayshort11                     EGEASR      ELQESSLEACRLVVDQQL   LPWSTGLQMRCCQQLR     CRPVALSQVAR

Ay        QTAVPPKGGSFYPRETTPLQQLQQGIFGGTSSQTVQGYYPSVISPQQGSYYPGQASPQQPGKWKELGQGQQGYYPTSLQQPGQGQQGYY
Ay11      QTAVPPK       ETTPLQQLQQGIFGGTSSQTVQG
Ay47      QTAVPPKGGSFYPRETTPLQQLQQG                       SYYPGQASPQQPGK  ELGQGQQGYYPTSLQQPGQGQQGYY
Ayshort47     PPKGGSFYPRETTPLQQLQQG    TSSQTVQGYYPSVISPQQGSYYPGQASPQQPGKWKELGQGQQGYYPTSLQQPGQGQQGYY
Ayshort11               ETTPLQQLQQGIFGGTSSQTVQG

Ay        QTAVPPKGGSFYPRETTPLQQLQQGIFGGTSSQTVQGYYPSVISPQQGSYYPGQASPQQPGKWKELGQGQQGYYPTSLQQPGQGQQGYY
Ay11
Ay47      QTAVPPKGGSFYPRETTPLQQLQQG                       SYYPGQASPQQPGK  ELGQGQQGYYPTSLQQPGQGQQGYY
Ayshort47 QTAVPPKGGSFYPRETTPLQQLQQG                       SYYPGQASPQQPGK  ELGQGQQGYYPTSLQQPGQGQQGYY
Ayshort11

Ay        RTSLQQPGQGQQIGQWQQGYYPTSLQHPGQGQQPGQVQKIGQGQQSEKGQQLGQEQQIGQGQQPEQGQQPGQGQQGYYPTSLQQPGQGQ
Ay11
Ay47      RTSLQQPGQGQQIGQWQQGYYPTSLQHPGQGQQPGQVQKIGQGQQSEKGQQLGQ
Ayshort47 RTSLQQPGQGQQIGQWQQGYYPTSLQHPGQGQQPGQVQKIGQGQQSEK
Ayshort11 RTSLQQPGQGQQIGQWQQGYYPTSLQHPGQGQQPGQVQKIGQGQQSEK

Ay        QPGQWQQAGQGQQGYYPTSLQQSGQGQQGHYPASQHQPGQGQQGHHPASLQQSGQGQQGHHPASLQQPGQGKQTGQREQRQQPGQGQQT
Ay11
Ay47                                                            QGQQGHHPASLQQPGQGK
Ayshort47                                                              PASLQQPGQGK
Ayshort11

Ay        GQGQQPEQEQQPGQGQQGYYPTYLQQPGQGQQPEQWQQPGQGQQGHYPASLQQSGQGQQGHYPDSLQQPGQGQPGQMQQPGQGQQPEQE
Ay11
Ay47
Ayshort47
Ayshort11

Ay        QQPGQGQQGYYPTSPQQPGQGQQPGQGQQGHFPTSGQAQQPGQGQQIGQVQQLGQGQQGYYPTSLQQPGQEQQSGQGQQLGQGHQPGQG
Ay11
Ay47
Ayshort47
Ayshort11

Ay        QQSGQEQQGYDNPYHVSVEQQAASPKVAKAHHPAAQLPIMCQMEGGDALSASQ
Ay11
Ay47                                   AHHPAAQLPIMCQMEGGDALSASQ
Ayshort47
Ayshort11
```

Fig. S3. Alignment of amino acid peptides obtained through MS/MS analysis. Ay corresponds to the deduced protein of the accession PI427346. A11 and A47 correspond to PI427346 and PI538442. The sequences of N-terminal sequencing are highlighted in grey, cysteines in green and aspartic acid in blue.
